# Public Health Genomics education in post-graduate schools of hygiene and preventive medicine: a cross-sectional survey

**DOI:** 10.1186/1472-6920-14-213

**Published:** 2014-10-10

**Authors:** Carolina Ianuale, Emanuele Leoncini, Walter Mazzucco, Carolina Marzuillo, Paolo Villari, Walter Ricciardi, Stefania Boccia

**Affiliations:** Section of Hygiene, Institute of Public Health, Università Cattolica del SacroCuore, Largo Francesco Vito 1, 00168 Rome, Italy; Department of Sciences for Health Promotion, University of Palermo, Palermo, Italy; Department of Public Health and Infectious Diseases, Sapienza University of Rome, Rome, Italy

**Keywords:** Survey, Education, Public health, Genomics

## Abstract

**Background:**

The relevance of Public Health Genomics (PHG) education among public health specialists has been recently acknowledged by the Association of Schools of Public Health in the European Region. The aim of this cross-sectional survey was to assess the prevalence of post-graduate public health schools for medical doctors which offer PHG training in Italy.

**Methods:**

The directors of the 33 Italian public health schools were interviewed for the presence of a PHG course in place. We stratified by geographical area (North, Centre and South) of the schools. We performed comparisons of categorical data using the chi-squared test.

**Results:**

The response rate was 73% (24/33 schools). Among respondents, 15 schools (63%) reported to have at least one dedicated course in place, while nine (38%) did not, with a significant geographic difference.

**Conclusions:**

Results showed a good implementation of courses in PHG discipline in Italian post-graduate public health schools. However further harmonization of the training programs of schools in public health at EU level is needed.

**Electronic supplementary material:**

The online version of this article (doi:10.1186/1472-6920-14-213) contains supplementary material, which is available to authorized users.

## Background

Since the first definition of Public Health Genomics (PHG) as “a multidisciplinary field concerned with the effective and responsible translation of genome-based knowledge and technologies to improve population health”[1], the relevance of PHG education has been acknowledged elsewhere. In Europe, since 2008 the Public Health scientific community, starting from Public Health Training in the Context of an Enlarging Europe (PHETICE) project”[2], has widely accepted that education and training of public health professionals should include “not only five core components of public health (epidemiology, biostatistics, environmental health, health service administration or management, and social and behavioral sciences referring to disease prevention and health promotion), but also critical new areas like informatics, genomics, communication, community-based participatory research, global health, ethics and last but not least policy and law”[3]. This has been also acknowledged by the Working Group on Innovation and Good Practice in Public Health Education within the Association of Schools of Public Health in the European Region (ASPHER - http://www.aspher.org/), which included PHG within the European Core Competences for Public Health Professionals[4].

With the advent of personalized healthcare and prevention, there is a need to prepare health care providers and public health practitioners to participate in research, evaluation and decision-making related to the use of genetic and genomic information in public health programs[5–7]. A set of core competences in genetics health care for health professionals has been recently defined by the European Society of Human Genetics[8]. These competences should be: “identify individuals who may have or may carry a genetic condition; communicate information about genetics in an understandable, comprehensible and sensitive way, helping patients to make informed decisions and choices about their care; manage patients with genetic conditions, using accepted guidelines; obtain specialist help and advice on inherited conditions; coordinate care with other primary-care professionals, geneticists and other appropriate specialists; offer appropriate psychological and social support to patients and families affected by a genetic condition”.

Previous literature on the topic[9–11] reported that medical doctors in general are not currently prepared to properly understand the significance of genomics to guide prevention or medical decisions. There are no studies, however, specifically investigating PHG training courses among the post-graduate medical schools in public health. This prompted us to carry out a study to assess the prevalence of on-going or planned courses on PHG among the post-graduate schools of public health in Italy, where current national policy envisages such need[12].

## Methods

We carried out a cross-sectional survey to evaluate the presence of teaching courses on PHG among the post-graduate schools of public health in Italy. Public health post-graduate resident trainings in ‘igiene and medicina preventiva’ (hygiene and preventive medicine), have a duration of five academic years and can only be entered by medical doctors. Questionnaires were sent by email on October, 1st 2012, to the Directors of the 33 post-graduate schools of hygiene and preventive medicine in Italy. We sent two reminders to increase the response rate.

We designed a questionnaire which investigated the following: (1) presence of a dedicated course in PHG, defined as a course focusing on any aspect dealing with the translation of genome-based knowledge and technologies in population health (e.g. from basic knowledge in genetics, to the ethical, legal and social issues, and organizational and policy issues) during the training period; (2) their exact content and (3) duration; (4) the academic year(s) in which the teaching course(s) take place; (5) modalities of exams; if there were no courses in place, (6) whether there was the intention to introduce it in the near future, and (7) whether the university had a full tenure in genetics, to investigate the potential relevance of the academic ‘genomic environment’ on the presence of PHG courses in the surveyed universities. Additional file1 shows the structure of the questionnaire.

We also calculated the cumulative h-index of the full team of academic members working in the university institutes of genetics. We identified eligible academic members through search of the university web pages at the same time the survey has been carried out. H-indexes were extrapolated from the ISI-Web of Knowledge database for each lecturer.

Results were reported as prevalence proportions and stratified according to the geographic areas (North, South, or Centre of Italy) of schools. Comparisons of categorical data were performed using the Pearson’s chi-squared test. We analyzed data using Stata software (StataCorp. 2011. *Stata Statistical Software: Release 12*. College Station, TX: StataCorp LP).

## Results

The response rate to the survey was 73% (24/33). Among the 24 respondents, six (25%) were located in the North of Italy, eleven (46%) in the Centre, seven (29%) in the South (29%). Among the non responders, five schools (55%) were located in the North of Italy, two (23%) in the Centre and two (23%) in the South.

**Figure 1:**
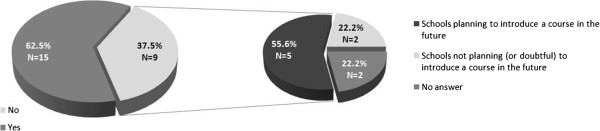
**Answers to questions concerning the presence of teaching course(s) on PHG among the post-graduate schools of hygiene and preventive medicine in Italy (n=24), and (if the course(s) was not in place) the intention to introduce such courses in the near future.**

Overall, 15 responding schools (63%) reported to have at least one course in place, while nine (38%) not. Four of these schools (27%) reported to have more than one PHG course during the five-years training.Of the nine schools that did not have a course on PHG, five (56%) were reportedly planning to introduce such courses in the near future; one school (11%) did not plan to introduce PHG teaching and one (11%) was unsure. The remaining two schools (22%) did not provide an answer to this question. Results are reported in the Figure 1.

Among the 15 schools with at least one course in place, the detailed contents were: genetic tests and basic knowledge in nine schools (60%); the impact of genomics in public health in seven schools (47%); multifactorial diseases and gene-environment interactions in six schools (40%); predictive medicine in five schools (33%) (some schools had more than one course).

Nine out of 15 schools (60%) organized PHG courses of a duration of 5–10 hours; three schools (20%) < 5 hours; and three schools (20%) > 10 hours.

Most schools (8, 53%) offered the PHG courses in the first academic year. One school (7%) offered the course in the second year, one in the third year (7%) and one (7%) in the fifth year. Four schools (26%) reported to have more than one course across the five years.

Residents were tested in PHG at the end of the course through an oral exams in most of the schools (12/15, 80%); the exam was written in two schools (13%); one school (7%) had no examination.

We found a significant difference in geographic distribution of the PHG courses: 100% of the schools in the North reported having a dedicated course in place, compared with 71% of the South and 36% of the Centre (*p* = 0.03).

Neither the presence of a full genetics tenure in the university, or the cumulative h-index of the genetics institute were significantly associated with the presence of a course on PHG in the surveyed universities (data not shown).

## Discussion

The integration of genomics in public health has the potential to improve the efficiency of health systems, but to reach this goal the development of suitable health care workforce is needed. The medical education in the genomic field is elsewhere acknowledged as of cornerstone importance to train health care practitioners in PHG[4, 7, 8].

The aim of our work was to survey the education on PHG among medical doctors during their training course period as public health specialists.

To our knowledge, this is the first survey in Europe among public health schools. The results show that the presence of a course in PHG was implemented among over 60% of the participating schools, independently from the relevance of the genetics schools at each university. Additionally, among those that did not have any course in place, more than 50% declared the intention to introduce it in the near future. As expected, there were differences among geographical areas, with the total of Northern respondent schools claiming to have a dedicated course, versus only the 36% of centre schools and the 73% of southern schools.

However, more than 60% of the surveyed schools reported that the focus of their courses was traditional genetics, with only 47% focusing on the impact of genomics in the provision of healthcare, which is more relevant to public health practitioners.

In our study we were unable to detect any influence of a full genetics tenure in the university. This was somewhat expected in view of the very recent introduction of a policy of PHG in Italy[12], released during the time of the survey has been conducted. We envisage that the awareness of the scientific community including Italian geneticists of the need of an integration of traditional genetics and public health will further increase in the near future.

In Italy, the *core curriculum* for the post-graduate schools of public health is currently under review. The present *curriculum* foresees one mandatory course in basic genetics at the 1^st^ year of training, and this is the place where half of the PHG lectures are offered. We believe, however, that in the forthcoming new *core curriculum* a larger and dedicated course should be included, in line with the recommendations of the ASPHER working group on Innovation and Good Practice in PH education[4] and an international working group on PHG[13].

Additionally, the testing method reported for most of the schools was oral exam, and this could be a problem in terms of objectivity of the results: we suggest the use of standard, written examinations, to better evaluate the achievement of core curriculum competences.

Caution is warranted in the interpretation of our results. Firstly, just above 70% of the schools responded to our survey: we believe this is a reasonable response rate, however some degree of selection bias might have been introduced, if schools that had courses in PHG were more likely to answer the survey as the topic may be of more interest to them. We might therefore have overestimated the proportion of schools where PHG courses are offered.

Secondly, the questionnaire was self-administered, hence inaccuracies might be present.

In conclusion, the results of this survey showed that a large proportion of post-graduate schools of hygiene and preventive medicine in Italy had PHG courses in place, with some geographic differences across the country.

## Conclusions

We believe that the implementation of PHG education among medical doctors is needed to ensure the sustainability for the National Health System, and in this context the formalization of mandatory courses in PHG in the *core curriculum* of the post-graduate schools of hygiene and preventive medicine has the potential to improve the policies in this field.

## Electronic supplementary material

Additional file 1: Table S1: Survey on Public Health Genomics education in post-graduate schools of hygiene and preventive medicine (Faculty of Medicine and Surgery). (PDF 54 KB)

## References

[1] Burke W, Khoury MJ, Stewart A, Zimmern RL, Bellagio Group (2006). The path from genome-based research to population health: development of an international public health genomics network. Genet Med.

[2] PHETICE (2008). Final Technical Report Covering the Period 01/04/2005 to 01/07/2008. Public Health Training in the Context of an Enlarging Europe.

[3] Boccia S, Khoury MJ, Zimmern R, Brand A, Brand H, Schroder P (2006). Public health genomics in Europe. Ital J Public Health.

[4] Birt CA, Foldspang A (2011). European Core Competences for Public Health Professionals (ECCPHP).

[5] Boccia S (2014). Why is personalized medicine relevant to public health?. Eur J PublicHealth.

[6] Mazzucco W, Ricciardi W, Boccia S (2012). Addressing the gap between genetics knowledge and clinical practice: a pilot study to implement genetics education among physicians in Italy. Ital J Public Health.

[7] Feero WG, Green ED (2011). Genomics education for health care professionals in the 21^st^ century. JAMA.

[8] Skirton H, Lewis C, Kent A, Coviello DA, and the members of Eurogentest Unit 6 and ESHG Education Committee (2010). Genetic education and the challenge of genomic medicine: development of core competences to support preparation of health professionals in Europe. Eur J Hum Genet.

[9] Bellcross CA, Kolor K, Goddard KA, Coates RJ, Reyes M, Khoury MJ (2011). Awareness and utilization of BRCA1/2 testing among U.S. primary care physicians. Am J PrevMed.

[10] Nippert I, Harris HJ, Julian-Reynier C, Kristofferson U, Ten Kate LP, Anionwu E, Benjamin C, Challen K, Schmidtke J, Nippert RP, Harris R (2011). Confidence of primary care physicians in their ability to carry out basic medical genetic tasks-a European survey in five countries-Part 1. J Community Genet.

[11] Marzuillo C, De Vito C, Boccia S, D’Addario M, D’Andrea E, Santini P, Boccia A, Villari P (2013). Knowledge, attitudes and behavior of physicians regarding predictive genetic tests for breast and colorectal cancer. PrevMed.

[12] Simone B, Mazzucco W, Gualano MR, Agodi A, Coviello D, Dagna Bricarelli F, Dallapiccola B, Di Maria E, Federici A, Genuardi M, Varesco L, Ricciardi W, Boccia S, GENISAP Network (2013). The policy of public health genomics in Italy. Health Policy.

[13] Boccia S, Mc Kee M, Adany R, Boffetta P, Burton H, Cambon-Thomsen A, Cornel MC, Gray M, Jani A, Maria Knoppers B, Khoury MJ, Meslin EM, Van Duijn CM, Villari P, Zimmern R, Cesario A, Puggina A, Colotto M, Ricciardi W: **Beyond public health genomics: proposals from an international working group.** *Eur J Public Health* [Epub ahead of print] 10.1093/eurpub/cku142 PMC4245010 25168910

[CR14] The pre-publication history for this paper can be accessed here:http://www.biomedcentral.com/1472-6920/14/213/prepub

