# Supplementary material for: Public Health Genomics education in post-graduate schools of hygiene and preventive medicine: a cross-sectional survey
Source: BMC Med Educ. 2014 Oct 10;14:213. doi: 10.1186/1472-6920-14-213 (PMC4283095; doi:10.1186/1472-6920-14-213)
Supplement: Supplementary file 1 — Additional file 1: Table S1: Survey on Public Health Genomics education in post-graduate schools of hygiene and preventive medicine (Faculty of Medicine and Surgery). (PDF 54 KB) [file 12909_2014_1097_MOESM1_ESM.pdf]

Additional file 1 Survey on Public Health Genomics education in post-graduate schools of hygiene and preventive medicine (Faculty of Medicine and Surgery)

Q1 - Is there a public health genomics course in place in the post-graduate programme?
- Yes ☐
- No ☐

If you answered yes to Q1:

Q2 - Which topic is covered in the course?
- Genetic test and basic concepts of genetics ☐
- The impact of genomics in public health ☐
- Other (please specify) ☐ ……………………………………..

Q3 - How long is the course (hours)?
- < 5 ☐
- 5 - 10 ☐
- > 10 ☐

Q4 - At what time (year) the course is delivered?
- First ☐
- Second ☐
- Third ☐
- Fourth ☐
- Fifth ☐

Q5 - Please specify the evaluation method
- Written exam ☐
- Oral exam ☐
- Exam not delivered ☐

If you answered no to Q1:

Q6 - Are you planning to offer a public health genomics course?
- Yes ☐
- No ☐
- Don't know ☐

Q7 - Does your university have a position for a full tenure in genetics?
- Yes ☐
- No ☐
